# Protocol for a controlled, randomized, blind, clinical trial to assess the effects of anodal transcranial direct current stimulation dorsolateral prefrontal cortex associated with balance training using games in the postural balance of older people

**DOI:** 10.12688/f1000research.25164.2

**Published:** 2021-02-23

**Authors:** Andre Issao Kunitake, João Carlos Ferrari Corrêa, Klaine Silva Nascimento, Bianca Barioni Cardoso de Oliveira, Natalia Maciel Muniz, Soraia Micaela Silva, Fernanda Ishida Corrêa

**Affiliations:** 1 Universidade Nove de Julho, Nove de Julho University, São Paulo, São Paulo, 01504-001, Brazil

**Keywords:** Elderly, postural balance, transcranial direct current stimulation, video game

## Abstract

**Aims:** This study aims to evaluate the additional effect of anodal transcranial direct current stimulation (a-tDCS) applied on dorsolateral pré-frontal cortex on training postural balance with the use of video games in the aged.

**Methods: **This is a blinded, randomized, controlled clinical trial protocol, with older people of both genders. Participants will be randomized into three training groups: Group 1 (videogame balance training), group 2 (videogame balance training associated with anodal tDCS), group 3 (videogame balance training associated with sham tDCS). The training will be carried out twice a week for four weeks, totaling eight sessions, and will be performed with the Nintendo Wii videogame console, using games that stimulate the postural balance associated with tDCS, with anode applied over the left dorsolateral prefrontal cortex and cathode on the contralateral supraorbital region at 2 mA for 20 minutes. The postural balance will be assessed using the Mini Test of the Balance Assessment System and posturography. Evaluations will be carried out before and after eight training sessions and 30 days after the end of treatment.

**Discussion:** Some studies show favorable results from the use of video games in improving postural balance in older people; however, their effect does not remain long-term. TDCS associated with other therapies can potentiate and prolong the effects of these therapies owing to its ability to stimulate neurotrophins important for neurogenesis, facilitating tasks that require attention, and helping to consolidate learning and memory. The effect of the two associated techniques on balance has not yet been tested in this population.

**Registration:** Brazilian Registry of Clinical Trials ID U1111-1213-4266; registered on 15 October 2018.

## Introduction

Aging is a physiological occurrence that has several consequences, including an increased risk of falls ^1, 2^. The incidence of falls increases proportionally to age, from 28% to 35% in the older people over 65 years and from 32% to 42% in those over 75 years. Falls can lead to musculoskeletal injuries, and in more severe cases, they can lead to death ^1, 3, 4^.

Preventive therapies for the risk of falls can be performed with the use of technological resources, such as videogames. Studies using videogames have shown positive effects in improving the postural control, balance, and functional capacity of the older people when practiced regularly ^5^. They have been widely used by therapists with high acceptance in clinical practice because they are motivating, challenging, and recreational, showing good evidence to improve postural control in the older people ^6– 9^.

Another technique that has aroused interest in clinical practice is transcranial direct current stimulation (tDCS), which consists of low-intensity current, and can be applied to the scalp by means of rubber electrodes, thus promoting changes in the potential of resting membrane, such as depolarization by the anode electrode or hyperpolarization by the cathode; modulating cortical excitability ^10, 11^. Anodal tDCS, when applied over the long term, can stimulate the brain-derived neurotrophic factor (BDNF), which is an important protein for stimulating neuroplasticity, improving attention, consolidating learning, and memory. Thus, its use has aroused interest for the older people, in order to reduce cognitive and motor declines ^12– 15^.

However, the protocols for the use of tDCS associated with tasks that involve balance, in order to improve balance are not yet defined, and are being applied in different dosages and locations ^16– 18^, therefore, further studies are necessary to verify the benefit of tCDS in association with other therapies. For that reason, the primary objective of this study is to assess whether tDCS can enhance and prolong the effects of videogame training on improving postural balance in the older people. Secondarily, check if there is a relationship between the response to training and the quality of life. This study hypothesizes that the association of tDCS with virtual reality therapy is more effective in improving balance in the older people than therapy with videogames alone, and that the addition of tDCS will prolong the effects of balance training.

## Methods

### Study design

**Figure 1.**
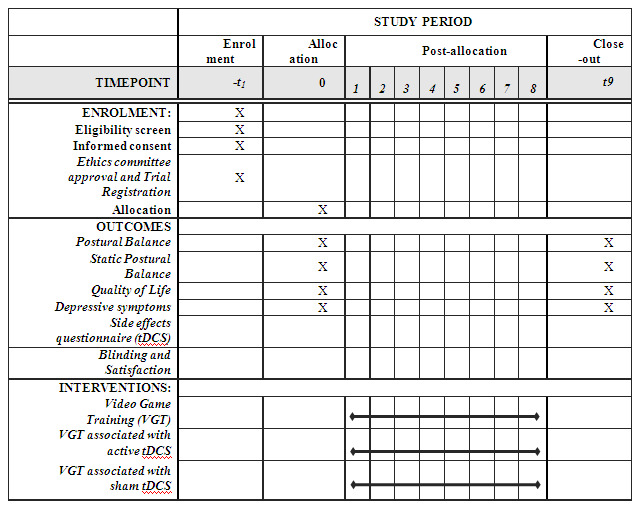
Study enrollment schedule, outcomes, and interventions. tDCS. transcranial direct current stimulation.

This is a protocol for a randomized, controlled blind, longitudinal clinical trial, as shown in Figure 1. This project will be carried out at Nove de Julho University in São Paulo, Brazil.

### Eligibility criteria

The inclusion criteria are people between 60 and 80 years old, of both genders, able to stand or walk without the aid of auxiliary devices, with reduced balance (score ≤25 points) evaluated using the Mini-BESTest ^19^. Exclusion criteria are cognitive impairment (scores ≤14 points) corrected for education, measured by the Mini-Mental State Examination (MMSE) ^20^, presence of lower limb fractures or amputations, neurological diseases, cardiovascular diseases that limit the performance of exercises, untreated acute and chronic respiratory diseases and the presence of pain that limits movement; the presence of contraindications to the use of tDCS.

### Consent to participate

The informed consent form will be explained to all participants. The volunteers who participate in this study, must sign the form (supplementary file 1), which guarantees the secrecy and confidentiality of data, free access to the final data, explanations of any kind related to the study and possible compensation for those that suffer from participation in the study.

### Ethics approval

The protocol was approved in May 2018 by Human Research Ethics Committee of the University Nove de Julho, São Paulo, Brazil (Opinion number 2.962.837), and Brazilian Clinical Trials Registry (ReBec), number: U1111-1213-4266, in accordance with Resolution 466/12 of the National Health Council of Brazil.

### Recruitment and allocation

Participants in this study will be community elders elected according to the criteria established for allocation. They will be randomized into 3 groups:

Group 1: Control Group (Video game balance training);

Group 2: Anodal group (Video game balance training associated with anodal tDCS);

Group 3: Sham Group (Video game balance training associated with sham tDCS).

A researcher not involved in evaluations or training will be responsible for allocating participants via www.randomization.com.

### Sample size

The sample size was obtained using G*Power 3.0.10 software, based on the outcomes from a pilot study. The calculation was carried out by the two-way repeated measures analysis of variance (ANOVA), considering the mean value (before and after training, and 30-day follow-up) for the control groups (25.46) active (27.46) and sham (24.66) and the pooled standard deviation (SD _pooled_) (2.89), with α = 0.05, β = 0.2 (80% of power) and the effect size of 0.40. A total of 15 individuals were determined to be required for each group (total sample: 45 individuals). Considering possible dropouts and to ensure a sample size that will demonstrate the effect of the intervention, the sample will be expanded by 25%, resulting in 19 individuals in each group, thus totalling 54 participants.

### Outcome assessments

All evaluations will be carried out on three occasions: pre-intervention, after eight treatment sessions and 30 days after the end of the training (follow-up). The training will be held twice a week, for 4 weeks, totalling 8 sessions.

### Primary outcomes

***Postural balance***. The assessment of postural balance will be performed by the Mini BESTest Scale, which consists of 14 functional tasks, such as sitting and getting up from a chair, standing up, balancing on tiptoes, and on one foot, overcoming walking obstacles, and double activities task ^21^.

Performance can range from 0 to 28 points. Each test can be performed for up to three attempts and the best result will be obtained. If adaptations are necessary to accomplish this, a point will be deducted from the maximum score obtained. This is a very reliable instrument, with an intraclass correlation (ICC) of 0.84) ^22^.

### Secondary outcome

***Static postural balance***. Posturography data will be collected using the Wii Balance Board (Nintendo, Kyoto, Japan), which is a validated instrument for posturography evaluation ^23^. The software for postural assessment is available at http://www.rehabtools.org/sway.html.

The evaluation protocol will be performed in two conditions, standing with eyes open and then with eyes closed. These two postures are reliable for measuring body sway, with eyes open with (ICC: 0.77) and with eyes closed (ICC: 0.89) ^23^.

To standardize the collections, the initial position of the feet on the evaluation platform will be marked and the same measurement repeated in all evaluations. The tests will last 1 minute, the initial 30 seconds will be to establish the patient’s suitability and the final 30 seconds will be to collect the posturography data.

***Quality of life (QOL)***. QOL will be measured by the World Health Organization’s Quality of Life Instrument, Bref version ^24^, which is composed of 26 questions, scored from 1 to 5, with the worst and the best scores being 26 and 130, respectively. In studies with the older people, it showed a high degree of reliability to mediate quality of life in the domain of physical capacity (0.89), psychological well-being (0.95), social relationships (0.81), and the environment (0,93).

### Potential confusion factors

***Symptoms of depression***. The symptoms of depression will be evaluated and classified according to severity, using the Beck Depression Inventory (BDI), a questionnaire composed of 21 questions, which can be self-applied. Scores from 0 to 10 indicate an absence of depression; 11 to 18 indicate mild depression; 19 to 29 indicate moderate depression and 30 to 63 is considered severe depression ^25^. In studies with the older people, the BDI score shows a degree of reliability r = 0.78 ^26^. Subsequently, it will be assessed whether the emotional state interfered with the results.

### The use of medication

The information related to continuous-use medication will be monitored through the application of a questionnaire prepared by the author. Such information will be used to characterize the sample.

### Intervention procedures

***Video game Balance training***. The balance training sessions will be performed using only the video games in group 1 (control), group 2 will be the balance training with the video games associated with the anodal tDCS and group 3 will be the balance training with the video games associated with the sham tDCS. In this study, a control group was added that performed the same training as the other groups. However, using only the video game. The justification for this was to verify if only the balance training with video game is efficient and if the tDCS additional can potentiate the training results, making the outcomes more effective. The sham group was used to verify if the response to balance training with video game, can be influenced by some placebo effect.

Video game training will be carried out with the Nintendo Wii and Wii Fit Plus, placed in a slide projector to enlarge the image. The sequence of games will always be the same, and the participant should play an average of 5 to 7 minutes each game, passing the stage according to their learning.

The sessions will be held twice a week, for four weeks, totalling eight sessions. Each session will last 20 minutes. The selected games are table tilt, penguin slide and ski slalom, which will be performed standing on the Wii Board Balance and require movements that stimulate balance, such as the anterior, posterior and lateral tilt of the body, without moving ( Figure 1).

### tDCS

tDCS will be performed with a tDCS device, the NeuroConn DC_STIMULATOR PLUS, from Germany, by means of two non-metallic surface electrodes, cathode 35 cm ^2^ (5 × 7 cm) and anode 25 cm ^2^ (5 × 5 cm), both wrapped in sponges moistened in a saline solution.

**Figure 2.**
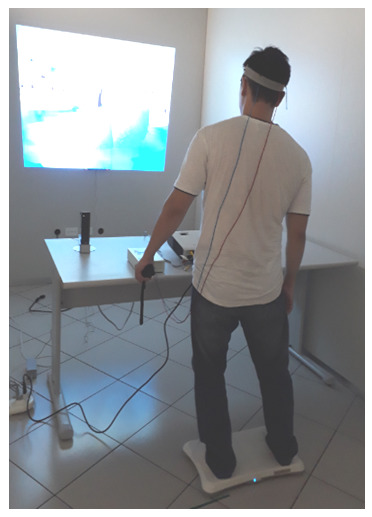
Positioning the participant on the board for the execution of the therapy with *Nintendo Wii* associated with active stimulation or sham. Source: author’s own, consent received for publication.

The intensity will be 2mA with a 20-second ramp up and down for a 20-minute period of stimulation. The montage will be anodal, unbalanced bilateral bipolar, anode positioned over the dorsolateral prefrontal cortex of the dominant hemisphere (F3 or F4) and the cathode electrode on the supraorbital region contralateral to the anode ( Figure 2), according to the criteria of the 10–20 electroencephalogram system ^27^. The application of the sham tDCS will occur in the same way, with the electrodes in the same positions as the anodal tDCS group, keeping working for 30 seconds.

### Assessment of the potential adverse effects

At the end of each session, a questionnaire on the adverse effects of tDCS will be applied to the participant ^24^ (see Extended data, Assessment of tDCS Adverse Events form).

### Blinding

The NeuroConn DC-STIMULATOR PLUS device has settings that allow the selection of the anodal stimulation or sham mode, by inserting codes. A researcher not involved in the procedures will program the equipment with the code to which the individual will be allocated. The stimulus mode will not be perceived by the external (supplementary functioning of the device, therefore, neither the researcher who will apply the intervention nor the individual will know what treatment will be applied (double-blind).

Participants in the group that will only perform training with video game will not be blind to treatment; however, a researcher will be responsible for the exclusive training of this group and will not be aware of the procedures with the tDCS of the other groups. All assessments will be made by researchers who have not participated in the training of the older people, and will, therefore, be blind to the intervention. A blinding questionnaire will be applied to guarantee its reliability and satisfaction with the treatment received (supplementary files 3 and 4), without the researcher knowing the training carried out by the participant.

### Statistical analysis

All statistical tests will be performed using SPSS (V22, IBM Corporation, New York, USA). Initially, we will perform the test of normality of the sample using the Shapiro-Wilk test, considering the significance level defined as a value of α < 0.05. The parametric data will be expressed as a mean ± SD (standard deviation), and the nonparametric data as a median (IQR); the categorical data will be described as absolute values and as a percentage of the total sample.

The variables of the data of the Mini-BESTest, Posturography, obtained pre-intervention, post-intervention and 30-day follow-up for the three groups (control, active and sham) will be calculated by the two-way repeated measures analysis of variance (ANOVA) for the parametric data and the Friedman’s test for the nonparametric data.

Finally, to verify whether there is a correlation between BESTest values and the symptoms of depression and quality of life, Pearson’s R correlation (parametric) or Spearman correlation (nonparametric) for the three groups in three evaluated times will be performed, verifying the degree of correlation (r) and its significance (p).

### Trial status

The protocol was approved in May 2018 by the Brazilian Clinical Trials Registry (ReBec) U1111-1213-4266. Submission of the manuscript occurred after this period, with the collection taking place and the completion is expected to happen in December 2020. The outcomes of this study will be subsequently published in a journal of interest.

## Discussion

The incidence of falls has become an aggravating problem with the increase in the number of older people and life expectancy. The costs of falling are also high; prevention is an effective and inexpensive alternative when compared to any other procedure ^28, 29^.

Preventive therapies can be performed with the use of video games, since they can be used to improve postural control and balance in the older people, helping to prevent falls. Besides, they are well accepted for being motivating and recreational and can be easily performed at home ^7– 10^.

However, there are several types of training protocols, which can vary from 4 to 20 weeks ^22^. For some older people, this long process of therapy can be tiring; however, for the consolidation of learning and memory to occur, repetitive training is necessary. According to 29, the learning and memory process is directly related to the number of repetitions performed by the older people, with a better effect in the long run. In this sense, the simultaneous application of tDCS can be a resource that can assist in this process of consolidating learning, improving attention for its ability to stimulate neuroplasticity. For the elderly, it can be an important tool in the rehabilitation process, as it is known that, with advancing age, they present cognitive and motor decline ^11– 14^.

However, one of the limitations of using tDCS for the older people is that few studies have investigated its effects on this population; some showing positive effects ^16, 18, 30, 31^, while others do not ^17, 32, 33^. Protocols combining the two resources have already been carried out in children with cerebral palsy ^34^, Parkinson’s disease ^35^, spinal cord injury ^36^ and stroke victims ^37, 38^. However, studies that associated tDCS and video games in the older people was not found.

## Conclusion

This article presents a randomized, controlled and blind protocol developed to show the effect of the combination of transcranial direct current stimulation (tDCS) associated with training using video games in the older people. The outcomes obtained will be published and their evidences may contribute for new training alternatives in the older people.

## Data availability

### Underlying data

No underlying data are associated with this article.

### Extended data

Harvard Dataverse: A PROTOCOL OF A CONTROLLED, RANDOMIZED, BLIND, CLINICAL TRIAL, TO CHECK THE EFFECTS OF ANODAL TRANSCRANIAL DIRECT CURRENT STIMULATION (tDCS) ASSOCIATED WITH BALANCE TRAINING USING GAMES IN THE POSTURAL BALANCE OF OLDER PEOPLE. https://doi.org/10.7910/DVN/OJBWHJ

File ‘Suplementary File.docx’ contains the following extended data:

- Model informed consent form.
- Assessment of tDCS Adverse Events form.
- Blinding questionnaire.
- Satisfaction questionnaire.

### Reporting guidelines

Harvard Dataverse: SPIRIT checklist for ‘Protocol for a controlled, randomized, blind, clinical trial to assess the effects of anodal transcranial direct current stimulation associated with balance training using games in the postural balance of older people’. https://doi.org/10.7910/DVN/OJBWHJ

Data are available under the terms of the Creative Commons Zero “No rights reserved” data waiver (CC0 1.0 Public domain dedication).
