# Supplementary material for: Protocol for a controlled, randomized, blind, clinical trial to assess the effects of anodal transcranial direct current stimulation dorsolateral prefrontal cortex associated with balance training using games in the postural balance of older people
Source: F1000Res. 2021 Feb 23;9:1018. Originally published 2020 Aug 21. [Version 2] doi: 10.12688/f1000research.25164.2 (PMC7713885; doi:10.12688/f1000research.25164.2)
Supplement: Peer review history

# Peer review: Reviewer response for version 2

**DOI:** 10.5256/f1000research.47058.r80073

**Published:** 2021-02-23

**Authors:** Rodrigo Vitório

**Affiliations:** 1 Department of Neurology, Oregon Health and Science University, Portland, OR, USA

The authors have addressed all my comments in the revised version.

Is the study design appropriate for the research question?

Yes

Is the rationale for, and objectives of, the study clearly described?

Yes

Are sufficient details of the methods provided to allow replication by others?

Yes

Are the datasets clearly presented in a useable and accessible format?

Not applicable

Reviewer Expertise:

Movement Science and Neuroscience, with experience in conducting clinical trials and reporting their findings.

I confirm that I have read this submission and believe that I have an appropriate level of expertise to confirm that it is of an acceptable scientific standard.

# Peer review: Reviewer response for version 1

**DOI:** 10.5256/f1000research.27770.r74514

**Published:** 2020-12-01

**Authors:** Rodrigo Vitório

**Affiliations:** 1 Department of Neurology, Oregon Health and Science University, Portland, OR, USA

This interesting ongoing randomized controlled trial examines whether anodal tDCS applied over the dorsolateral prefrontal cortex can enhance and/or prolong the effects of videogame training on balance in older adults. The authors report existing approval by the local ethics committee and ongoing training/intervention and data collection. The study is registered at ensaiosclinicos.gov.br. Methods are sound. I have only a few suggestions that I believe will make the manuscript clearer. 

- Please mention stimulation type (i.e., "anodal" or "anodic") and targeted area (i.e., dorsolateral prefrontal cortex) in the title and aims (abstract and main text).
- Please clarify why the study includes a "control group" (no electrodes positioning) and a "sham group" (placebo tDCS). 
- Please provide further details about the sham tDCS (e.g., will any current intensity be applied? For how long? Etc.)
- Please avoid the use of “elderly”. Consider using “older adults/people” throughout the entire manuscript.
- The primary/secondary outcome measures should be explicitly identified. 

Is the study design appropriate for the research question?

Yes

Is the rationale for, and objectives of, the study clearly described?

Yes

Are sufficient details of the methods provided to allow replication by others?

Yes

Are the datasets clearly presented in a useable and accessible format?

Not applicable

Reviewer Expertise:

Movement Science and Neuroscience, with experience in conducting clinical trials and reporting their findings.

I confirm that I have read this submission and believe that I have an appropriate level of expertise to confirm that it is of an acceptable scientific standard.

# Response

**Published:** 2020-12-21

**Authors:** Andre Issao Kunitake

**Affiliations:** Universidade Nove de Julho, Brazil

Dear reviewer. We appreciate your time reading our study and making your considerations. I adapted the article taking into account everything that was requested, to enrich and make this article more quality.

 Best regards

# Peer review: Reviewer response for version 1

**DOI:** 10.5256/f1000research.27770.r71407

**Published:** 2020-11-02

**Authors:** Fernando Zanela da Silva Arêas

**Affiliations:** 1 Center of Health Sciences, Discipline of Physical Therapy, Federal University of Espírito Santo (UFES), Vitória, Brazil

The paper is well delineated, and well written - it is clear, objective and relevant. The protocols follow all the ethics criteria for reproduction. Also, the literature does not have works that investigate the approach with this methodologic quality. 

 Does the project have some finance?

 This is a good project. I recommend indexing.

Is the study design appropriate for the research question?

Yes

Is the rationale for, and objectives of, the study clearly described?

Yes

Are sufficient details of the methods provided to allow replication by others?

Yes

Are the datasets clearly presented in a useable and accessible format?

Yes

Reviewer Expertise:

Neurorehabilitation, neuromodulation, traumatic brain inury

I confirm that I have read this submission and believe that I have an appropriate level of expertise to confirm that it is of an acceptable scientific standard.
